# Supplementary material for: Distinct Target-Specific Mechanisms Homeostatically Stabilize Transmission at Pre- and Post-synaptic Compartments
Source: Front Cell Neurosci. 2020 Jun 26;14:196. doi: 10.3389/fncel.2020.00196 (PMC7333441; doi:10.3389/fncel.2020.00196)
Supplement: Supplementary file 1 [file Data_Sheet_1.PDF]

Supplementary information for

Distinct target-specific mechanisms homeostatically stabilize transmission at pre-and post-synaptic compartments

Pragya Goel, Samantha Nishimura, Karthik Chetlapalli, Xiling Li, Catherine Chen, and Dion Dickman

Correspondence: Dion Dickman

E-mail: dickman@usc.edu

This supplemental file includes

Figures S1 to S3

Tables S1 to S2

SI References

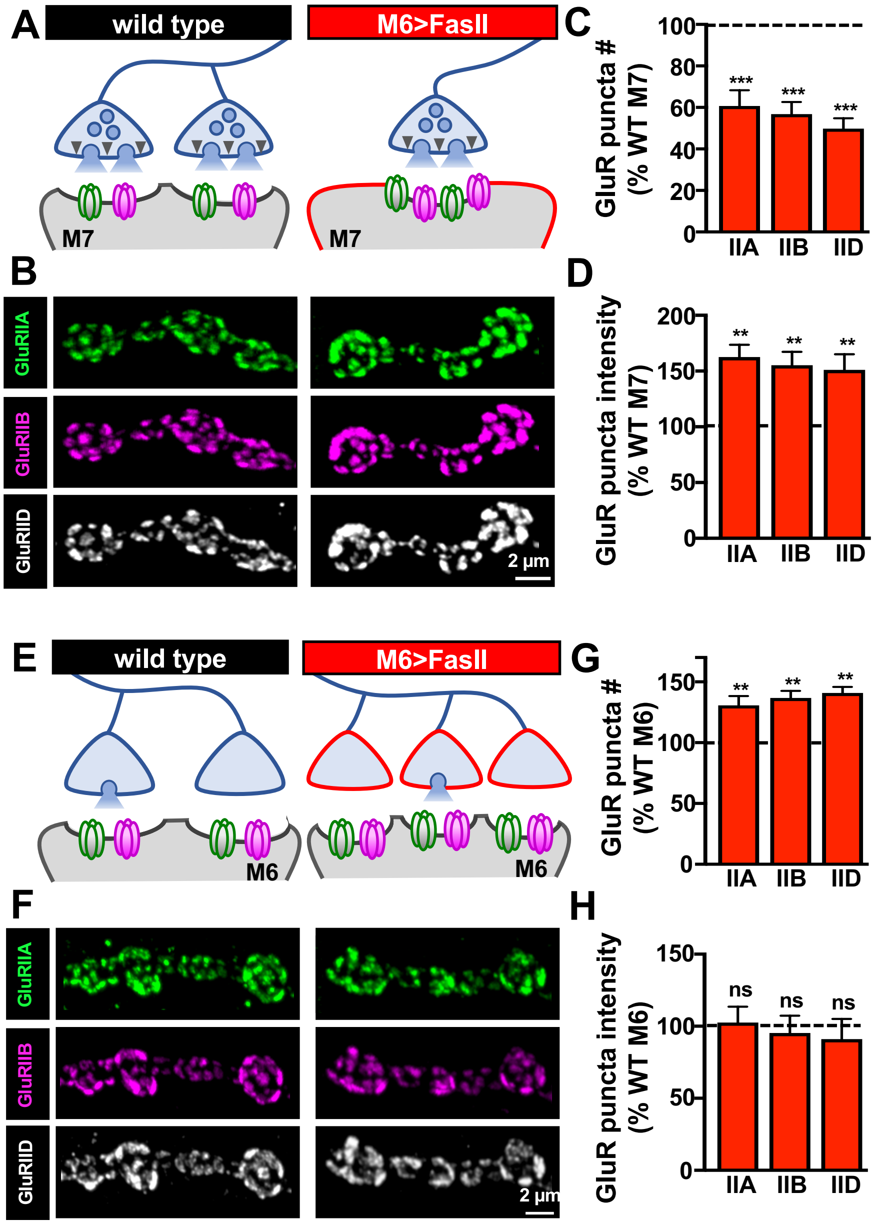
A
wild type
M6>FasII
M7
M7
B
GluRIIA
GluRIIB
GluRIID
2 µm
C
GluR puncta #
(% WT M7)
100
80
60
40
20
0
***
***
***
IIA
IIB
IID
D
GluR puncta intensity
(% WT M7)
200
150
100
50
0
**
**
**
IIA
IIB
IID
E
wild type
M6>FasII
M6
M6
F
GluRIIA
GluRIIB
GluRIID
2 µm
G
GluR puncta #
(% WT M6)
150
100
50
0
**
**
**
IIA
IIB
IID
H
GluR puncta intensity
(% WT M6)
150
100
50
0
ns
ns
ns
IIA
IIB
IID

Figure S1: Postsynaptic GluR levels are enhanced at hypo-innervated targets but do not change at hyper-innervated targets. (A) Schematic illustrating enhanced levels of both GluRIIA- and GluRIIB-containing receptors on hypo-innervated muscle 7 in M6>FasII. **(B)** Representative images of individual boutons at NMJs immunostained with antibodies that recognize the postsynaptic GluR subunits GluRIIA, GluRIIB, and GluRIID. Quantification of the indicated GluR puncta number **(C)** and intensity **(D)** normalized to wild type muscle 7 reveals a significant reduction in GluR puncta number but increase in puncta intensity at hypo-innervated muscle 7. **(E)** Schematic illustrating that no changes in postsynaptic GluR levels are induced at hyper-innervated NMJs. **(F)** Representative images of individual boutons in the indicated genotypes at muscle 6 NMJs immunostained with anti-GluRIIA, -GluRIIB, and -GluRIID. **(G)** Quantification of GluR puncta number normalized to wild type muscle 6 demonstrates a significant increase in M6>FasII. **(H)** The total sum intensity of individual GluRIIA, GluRIIB, and GluRIID puncta on muscle 6 NMJs is unchanged in M6>FasII compared to wild type. Error bars indicate ±SEM ($n \geq 9$; one-way ANOVA; Table S2). **$p<0.01$; ***$p<0.001$; ns=not significant.

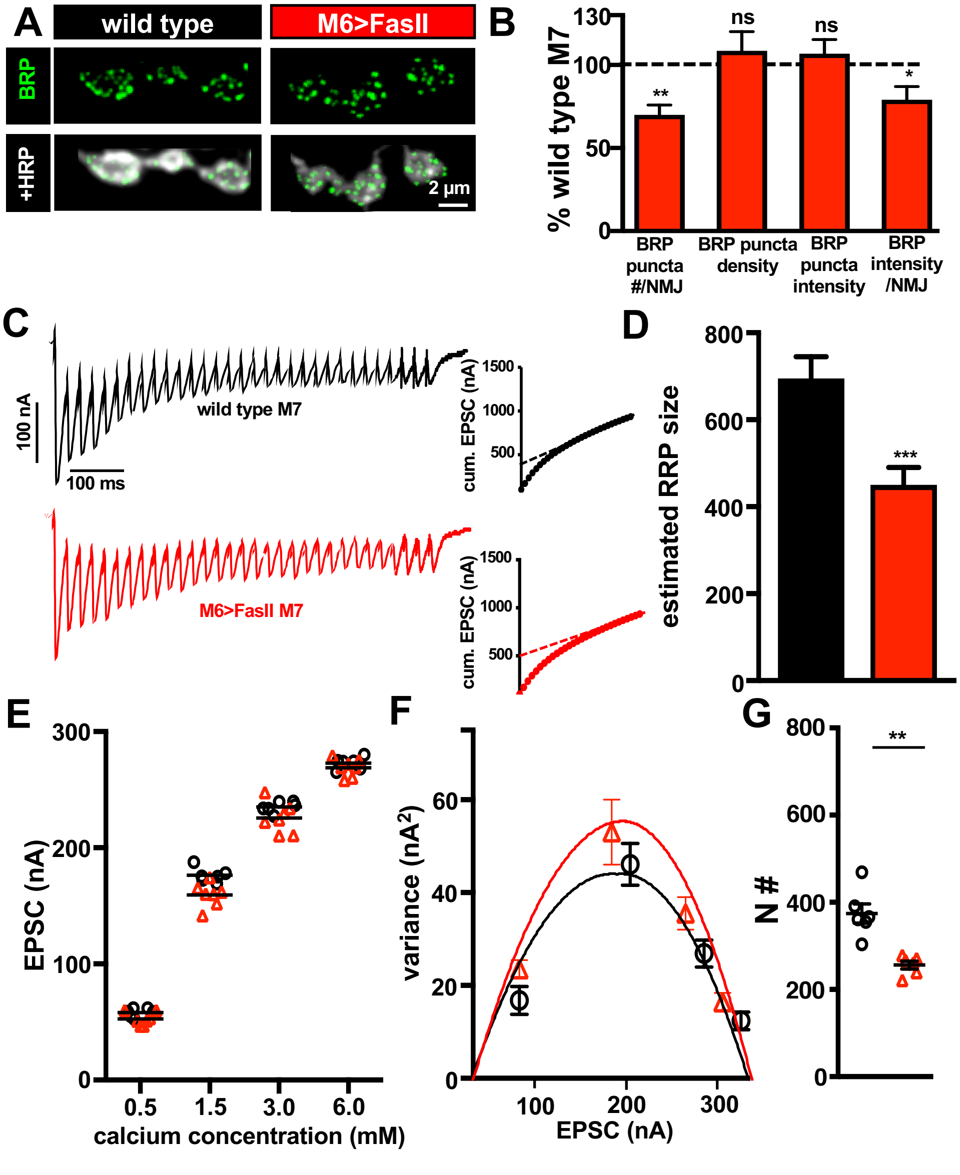
A
wild type
M6>FasII
BRP
+HRP
2 µm
B
% wild type M7
130
100
50
0
**
ns
ns
*
BRP puncta #/NMJ
BRP puncta density
BRP puncta intensity
BRP intensity /NMJ
C
100 nA
100 ms
wild type M7
M6>FasII M7
cum. EPSC (nA)
1500
1000
500
D
estimated RRP size
800
600
400
200
0
***
E
EPSC (nA)
300
200
100
0
0.5
1.5
3.0
6.0
calcium concentration (mM)
F
variance (nA²)
60
40
20
0
100
200
300
EPSC (nA)
G
N #
800
600
400
200
0
**

Figure S2: No apparent changes in presynaptic function and the size, density or intensity of active zones are observed at hypo-innervated NMJs. (A) Representative images of individual boutons at muscle 7 NMJs in wild type and hypo-innervated M6>FasII immunostained with an antibody against the active zone scaffold BRP. **(B)** Quantification of BRP puncta number, density, and intensity on muscle 7 in M6>FasII normalized to wild-type values. A significant decrease in BRP puncta number but no change in density is observed. No significant change in the total intensity of each individual BRP puncta is observed (puncta sum intensity), while the total fluorescence intensity of all BRP puncta summed across the entire muscle 7 NMJ is reduced in hypo-innervated targets. **(C)** Representative EPSC traces of 30 stimuli at 3 mM extracellular Ca^{2+} during a 60 Hz stimulus train recorded in two-electrode voltage clamp (TEVC) configuration in the indicated genotypes. Graphical insets show the average cumulative EPSC plotted as a function of time. A line fit to the 18th-30th stimuli was back-extrapolated to time 0. **(D)** The size of the RRP is reduced by a proportion similar to the reduction in quantal content on muscle 7 in M6>FasII. **(E)** Scatter plot EPSC distribution of recordings from muscle 7 in wild type (black circles) and M6>FasII (red triangles) in the indicated extracellular Ca^{2+} concentrations. **(F)** Variance-mean plots for the indicated genotypes. Variance was plotted against the mean amplitude of 30 EPSCs recorded at 0.2 Hz from the Ca^{2+} concentrations detailed in (D)**.** Lines are the best fit parabolas to the data points. **(G)** Estimated number of functional release sites (N #) obtained from the variance-mean plots in (H) showing a significant reduction on muscle 7 of M6>FasII compared to wild type muscle 7. Error bars indicate ±SEM (n≥9; one-way ANOVA; Table S2). $^{**}p<0.01$; $^{***}p<0.001$; ns=not significant.

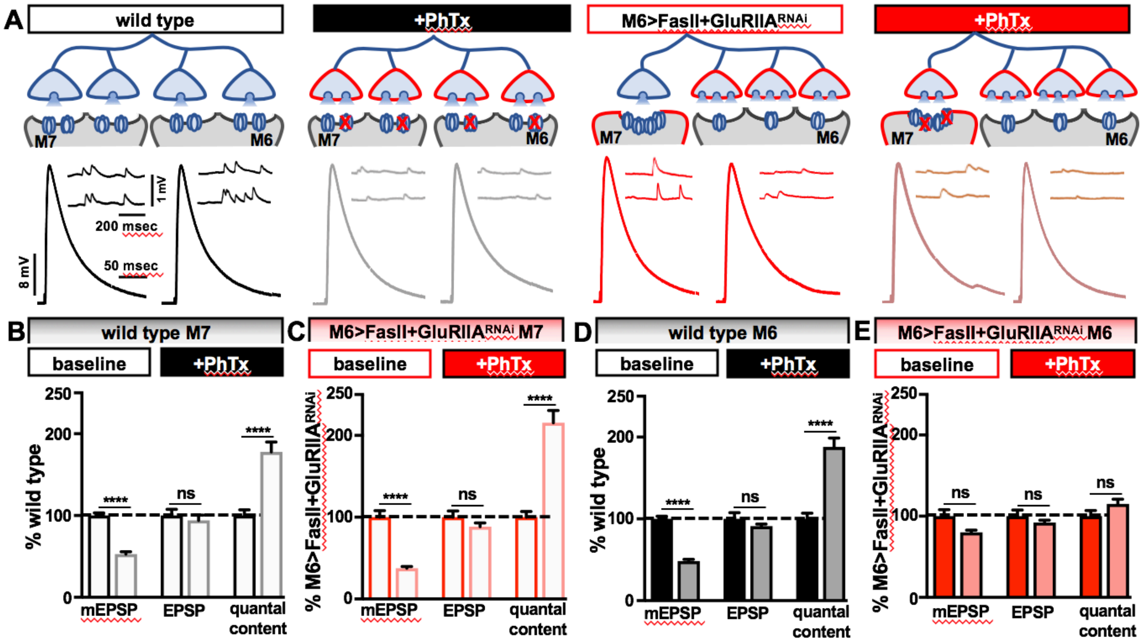

A
wild type
+PhTx
M6>FasII+GluRIIA^RNAi
+PhTx
M7
M6
1 mV
200 msec
50 msec
8 mV
B
wild type M7
baseline
+PhTx
% wild type
mEPSP
EPSP
quantal content
C
M6>FasII+GluRIIA^RNAi M7
% M6>FasII+GluRIIA^RNAi
D
wild type M6
E
M6>FasII+GluRIIA^RNAi M6
ns
****

Figure S3: PHP can be rapidly expressed at hypo-innervated targets to balance synaptic strength. (A) Schematics and representative traces illustrating the acute application of PhTx on wild type and M6>FasII+GluRIIA^{RNAi} NMJs. Note that PhTx application reduces mEPSP amplitudes on hypo-innervated targets, yet EPSP amplitudes remain similar to baseline values because of a homeostatic increase in presynaptic neurotransmitter release, indicating robust PHP expression. **(B,C,D,E)** Quantification of mEPSP amplitude, EPSP amplitude and quantal content values normalized to baseline values (wild type muscle 7 in (B) and 6 in (D); M6>FasII+GluRIIA^{RNAi} muscle 7 (C) or 6 (E)). Error bars indicate ±SEM (n≥8; Student’s t-test; Table S2). ****p<0.0001; ns=not significant.

Table S1. REAGENTS AND RESOURCES TABLE

Antibodies			
**REAGENT/RESOURCE**	**SOURCE**	**IDENTIFIER**	**DILUTION**
Mouse anti-Synapsin (3C11)	Developmental Studies Hybridoma Bank (DSHB)	AB_2313867	1:10
Tetramethylrhodamine (TRITC)-conjugated phalloidin (R415)	Thermo Fisher Scientific	41-6559-05	1:1000
Mouse anti-Bruchpilot (nc82)	DSHB	AB_2314866	1:100
Guinea pig anti-vGlut	(Goel and Dickman, 2018)	N/A	1:2000
Mouse anti-GluRIIA (8B4D2)	DSHB	AB_528269	1:50
Affinity-Purified Rabbit anti-GluRIIB	(Perry et al., 2017)	N/A	1:2000
Guinea pig anti-GluRIID	(Kikuma et al., 2017)	N/A	1:1000
DyLight 405-conjucated secondary antibodies	Jackson ImmunoResearch Laboratories, Inc	706-475-148	1:400
Alexa Fluor 488-conjugated secondary antibodies	Jackson ImmunoResearch Laboratories, Inc	706-545-148, 715-545-150, 711-545-152	1:400
Cy3-conjugated secondary antibodies	Jackson ImmunoResearch Laboratories, Inc	706-165-148, 715-165-150, 711-165-152	1:400
Alexa Fluor 647 conjugated Goat anti-Horseradish Peroxidase	Jackson ImmunoResearch Laboratories, Inc	123-605-021	1:200

Experimental Models: Fly Lines		
**REAGENT/RESOURCE**	**REFERENCE**	**SOURCE**
H94-Gal4	(Davis et al., 1997)	Brain McCabe<br>Ecole polytechnique federale de Lausanne
UAS-FasII-PEST+	(Davis et al., 1997)	C. Andrew Frank<br>University of Iowa
GluRIIA^{sp16}	(Petersen et al., 1997)	Bloomington Drosophila Stock Center (BDSC #64202)
Cac$^{sfGFP-N}$	(Gratz et al., 2019)	Kate O'Connor Giles<br>Brown University
UAS-GluRIIA^{RNAi} (p{TRiP.JF02647}attP2)	(Li et al., 2018)	Bloomington Drosophila Stock Center (BDSC #27497)
Tub-FRT-STOP-FRT-Gal4,UAS-FLP,UAS-CD8-GFP	(Roy et al., 2007)	Brain McCabe<br>Ecole polytechnique federale de Lausanne

Supplemental References:

Davis, G.W., C. Schuster, and C.S. Goodman. 1997. Genetic analysis of the molecular mechanisms controlling target selection: target derived Fasciclin II regulates the pattern of synapse formation. *Neuron*. 19:561-573.

Goel, P., and D. Dickman. 2018. Distinct homeostatic modulations stabilize reduced postsynaptic receptivity in response to presynaptic DLK signaling. *Nat Commun*. 9:1856.

Gratz, S.J., P. Goel, J.J. Bruckner, R.X. Hernandez, K. Khateeb, G. Macleod, D. Dickman, and K.M. O'Connor-Giles. 2019. Endogenous tagging reveals differential regulation of Ca2+ channels at single AZs during presynaptic homeostatic potentiation and depression. *J Neurosci*. 39:2416-2429.

Kikuma, K., X. Li, D. Kim, D. Sutter, and D.K. Dickman. 2017. Extended Synaptotagmin Localizes to Presynaptic ER and Promotes Neurotransmission and Synaptic Growth in Drosophila. *Genetics*. 207:993-1007.

Li, X., P. Goel, C. Chen, V. Angajala, X. Chen, and D. Dickman. 2018. Synapse-specific and compartmentalized expression of presynaptic homeostatic potentiation. *Elife*. 7:e34338.

Perry, S., Y. Han, A. Das, and D.K. Dickman. 2017. Homeostatic plasticity can be induced and expressed to restore synaptic strength at neuromuscular junctions undergoing ALS-related degeneration. *Human Mol Genet.* 26:4153-4167.

Petersen, S.A., R.D. Fetter, J.N. Noordermeer, C.S. Goodman, and A. DiAntonio. 1997. Genetic analysis of glutamate receptors in Drosophila reveals a retrograde signal regulating presynaptic transmitter release. *Neuron*. 19:1237-1248.

Roy, B., A.P. Singh, C. Shetty, V. Chaudhary, A. North, M. Landgraf, K. Vijayraghavan, and V. Rodrigues. 2007. Metamorphosis of an identified serotonergic neuron in the Drosophila olfactory system. *Neural Dev* 2:20.

Table S2. Absolute values for normalized data and additional statistics. The figure and panel, genotype, and conditions used are noted. For electrophysiological recordings, average values for mEPSP, EPSP, quantal content (QC), resting membrane potential, input resistance, number of data samples (n), p values, and significance are shown. For confocal imaging analysis, average values for intensity levels of indicated synaptic markers and other relevant parameters such as numbers and area are shown. Standard error values are noted in parentheses. Rows highlighted in blue are the respective controls or baseline values for the particular experiment being referenced.

Figure	Genotype	mEPSP (mV)	EPSP (mV)	QC	mEPSP (Hz)	bouton #/muscle	QC/ bouton	Resting Potential (mV)	Input Resistance (MΩ)	n	P value (significance) (mEPSP amp, EPSP, QC boutons, QC/bouton, mEPSP freq)
1D,E	*w^1118* (muscle 7)	0.965 (0.012)	35.37 (1.99)	36.65 (2.047)	2.545 (0.096)	39.75 (1.509)	0.922 (0.071)	-68.7 (1.412)	14.512 (1.216)	14	
1D,E	*w;H94-Gal4;UAS-FasII* (muscle 7)	1.329 (0.063)	32.08 (1.016)	24.061 (1.614)	1.815 (0.155)	21.81 (2.201)	1.038 (0.011)	-67.538 (1.409)	12.013 (0.819)	16	0.0055 (**), >0.9999 (ns), 0.0076 (**), <0.0001 (****), 0.5021 (ns), 0.0091 (**)
1D,E	*w^1118* (muscle 6)	1.018 (0.071)	34.29 (0.815)	33.683 (1.97)	2.977 (0.091)	58.88 (2.856)	0.572 (0.043)	-64.491 (1.221)	7.215 (0.237)	14	
1D,E	*w;H94-Gal4;UAS-FasII* (muscle 6)	1.021 (0.015)	30.84 (0.764)	30.271 (1.899)	3.009 (0.148)	80.14 (1.993)	0.377 (0.025)	-65.559 (1.720)	8.790 (0.504)	16	>0.9999 (ns), 0.9157 (ns), 0.9667 (ns), 0.0004 (***) 0.0083 (**), 0.9995 (ns)

Figure	Genotype	Failure rate (0.15 mM Ca^{2+})	Paired-pulse facilitation (PPF) rate (0.4 mM Ca^{2+})	Paired-pulse depression (PPD) rate (1.5 mM Ca^{2+})	n	P value (significance) (failure, PPF, PPD)
2B,D,F	*w^1118* (muscle 6)	57.51 (3.541)	140.44 (7.128)	69.55 (4.028)	9,9,7	
2B,D,F	*w;H94-Gal4;UAS-FasII* (muscle 6)	51.02 (5.014)	167.91 (10.102)	88.12 (7.662)	8,9,8	0.8813 (ns), 0.0081 (**), 0.0061 (**)

Figure	Genotype	Estimated mEPSP (mV)	Cumulative EPSC (nA)	Estimated RRP size	n	P value (significance) (mEPSC, cum. EPSC, RRP)
S2C,D	*w^1118* (muscle 7)	0.732 (0.027)	509.88 (40.128)	695.55 (47.828)	9	
S2C,D	*w;H94-Gal4;UAS-FasII* (muscle 7)	0.902 (0.014)	500.24 (42.102)	451.29 (40.662)	8	0.0071 (**), 0.8559 (ns), 0.0008 (***)
2G,H	*w^1118* (muscle 6)	0.781 (0.026)	877.97 (56.705)	1125.55 (105.711)	9	
2G,H	*w;H94-Gal4;UAS-FasII* (muscle 6)	0.827 (0.068)	1005.21 (63.031)	1215.67 (93.459)	8	0.6991 (ns), 0.9978 (ns), 0.8761 (ns)

Figure	Genotype	Functional release site number (N)	n	P value (significance)
S2G	*w^1118* (muscle 7)	468.86 (27.81)	6	
S2G	*w;H94-Gal4;UAS-FasII* (muscle 7)	320.3 (11.16)	6	0.0021 (**)
2K	*w^1118* (muscle 6)	500.97 (36.22)	9	
2K	*w;H94-Gal4;UAS-FasII* (muscle 6)	410.78 (36.38)	10	0.9711 (ns)

Figure	Genotype	BRP puncta #/NMJ	BRP puncta density (#/μm^2)	BRP puncta Intensity (% WT)	Total BRP intensity/M7 NMJ (% WT)	n	P value (significance) (BRP puncta #, density, puncta intensity, total intensity)
S2B	*w^{1118}* (muscle 7)	141.67 (11.57)	0.916 (0.014)	100 (7.526)	100 (6.664)	14	
S2B	*w;H94-Gal4;UAS-FasII* (muscle 7)	99.17 (5.851)	0.994 (0.111)	106.821 (8.451)	78.93 (8.003)	16	0.0049 (**), 0.8976 (ns), 0.6781 (ns), 0.0105 (*)
3D,E,F,G	*w^{1118}* (muscle 6)	261.44 (18.765)	0.974 (0.087)	100 (6.167)	100 (9.778)	14	
3D,E,F,G	*w;H94-Gal4;UAS-FasII* (muscle 6)	309.72 (20.949)	0.737 (0.062)	78.76 (7.098)	94.77 (9.175)	16	0.0205 (*), 0.0051 (**), 0.0084 (**), 0.8846 (ns)

Figure	Genotype	Cac puncta #/NMJ	Cac puncta density (#/μm^2)	Cac puncta intensity (% WT)	Total Cac intensity/M7 NMJ (% WT)	n	P value (significance) (Cac puncta #, density, puncta intensity, total intensity)
3D,E,F,G	*$cac^{sfGFP-N}$*(muscle 6)	132.44 (10.628)	0.881 (0.064)	100 (7.443)	100 (9.803)	13	
3D,E,F,G	*$cac^{sfGFP-N}$; H94-Gal4;UAS-FasII* (muscle 6)	141.68 (11.917)	0.629 (0.053)	81.97 (7.255)	92.88 (8.765)	12	0.6267 (ns), 0.0053 (**), 0.0075 (**), 0.9121 (ns)

Figure	Genotype	GluRIIA puncta #/NMJ	GluRIIB puncta #/NMJ	GluRIID puncta #/NMJ	n	P value (significance) (GluRIIA, GluRIIB, GluRIID)
S1C	*w^{1118}* (muscle 7)	164.63 (9.977)	155.53 (10.093)	168.21 (11.288)	9	
S1C	*w;H94-Gal4;UAS-FasII* (muscle 7)	92.9 (6.163)	85.133 (6.292)	72.66 (9.035)	10	0.0007 (***), 0.0005 (***), 0.0003 (***)
S1G	*w^{1118}* (muscle 6)	208.125 (14.109)	218.33 (13.751)	197.75 (13.607)	9	
S1G	*w;H94-Gal4;UAS-FasII* (muscle 6)	278.11 (20.109)	289.71 (22.892)	267.516 (19.955)	10	0.0049 (**), 0.0068 (**), 0.0071 (**)

Figure	Genotype	GluRIIA puncta intensity (%WT)	GluRIIB puncta intensity (%WT)	GluRIID puncta intensity (%WT)	n	P value (significance) (GluRIIA, GluRIIB, GluRIID)
S1D	*w^{1118}* (muscle 7)	100 (6.271)	100 (5.828)	100 (7.188)	9	
S1D	*w;H94-Gal4;UAS-FasII* (muscle 7)	162.54 (11.11)	155.21 (12.292)	151.09 (14.65)	10	0.0068 (**), 0.0059 (**), 0.0079 (**)
S1H	*w^{1118}* (muscle 6)	100 (4.769)	100 (6.715)	100 (5.407)	9	
S1H	*w;H94-Gal4;UAS-FasII* (muscle 6)	102.54 (10.98)	95.21 (13.03)	91.12 (13.955)	10	0.6991 (ns), 0.9978 (ns), 0.8761 (ns)

Figure	Genotype	mEPSP (mV)	EPSP (mV)	QC	GluRIIA intensity (%WT)	bouton #/muscle	Resting Potential (mV)	Input Resistance (MΩ)	n	P value (significance) (mEPSP, EPSP, QC, GluRIIA, bouton #)
4E	w^{1118} (muscle 7)	0.96 (0.012)	35.37 (1.99)	36.65 (2.047)	100 (4.961)	39.75 (1.509)	-68.7 (1.412)	14.512 (1.216)	14	
4E	*w;Tub-FRT-STOP-FRT-Gal4,UAS-FLP,UAS-CD8-GFP;H94-Gal4,nSyb-Gal80/UAS-FasII* (muscle 7)	1.369 (0.041)	33.19 (1.536)	24.24 (1.614)	168.15 (16.053)	18.75 (2.201)	-63.713 (2.009)	12.013 (0.819)	12	0.0002 (***), 0.9885 (ns), 0.0045 (**), 0.0041 (**) <0.0.0001 (****) [Compared to WT-muscle7]
4E	*w;Tub-FRT-STOP-FRT-Gal4,UAS-FLP,UAS-CD8-GFP;H94-Gal4,nSyb-Gal80/UAS-FasII;UAS-GluRIIA*^{RNAi} (muscle 7)	1.35 (0.039)	31.71 (1.991)	22.37 (1.211)	156.74 (21.191)	19.21 (0.945)	-65.51 (1.875)	15.51 (0.98)	11	>0.9999 (ns), >0.9999 (ns), 0.9976(ns), >0.9999 (ns), >0.9999 (ns)
4F	w^{1118} (muscle 6)	1.018 (0.071)	34.29 (0.815)	33.683 (1.97)	100 (5.543)	58.88 (2.856)	-64.491 (1.221)	7.215 (0.237)	14	
4F	*w;Tub-FRT-STOP-FRT-Gal4,UAS-FLP,UAS-CD8-GFP;H94-Gal4,nSyb-Gal80/UAS-FasII* (muscle 6)	0.97 (0.022)	32.94 (0.894)	33.95 (1.659)	100 (6.78)	72.44 (5.551)	-65.559 (1.720)	9.120 (0.414)	12	0.9786 (ns), >0.9999 (ns), >0.9999 (ns), 0.9997 (ns), 0.0045 (**) [Compared to WT-muscle6]
4F	*w;Tub-FRT-STOP-FRT-Gal4,UAS-FLP,UAS-CD8-GFP;H94-Gal4,nSyb-Gal80/UAS-FasII;UAS-GluRIIA*^{RNAi} (muscle 6)	0.46 (0.027)	31.94 (1.708)	66.54 (3.732)	10.79 (2.231)	70.99 (5.96)	-62.87 (1.591)	11.69 (1.065)	11	<0.0001 (****), 0.9996 (ns), <0.0001 (****), <0.0001 (****),>0.9999 (ns)

Figure	Genotype	PhTx	mEPSP (mV)	EPSP (mV)	QC	Resting Potential (mV)	Input Resistance (MΩ)	n	P value (significance) (mEPSP, EPSP, QC)
S3B	w^{1118} (muscle 7)	-	0.954 (0.028)	33.1 (1.547)	34.59 (2.292)	-64.31 (2.015)	12.35 (0.993)	9	
S3B	w^{1118} (muscle 7)	+	0.5 (0.026)	31.11 (2.15)	62.22 (4.193)	-66.55 (3.036)	10.95 (0.768)	9	<0.0001 (****), 0.9995 (ns), <0.0001 (****)
S3C	*w;Tub-FRT-STOP-FRT-Gal4,UAS-FLP,UAS-CD8-GFP;H94-Gal4,nSyb-Gal80/UAS-FasII;UAS-GluRIIA*^{RNAi} (muscle 7)	-	1.35 (0.039)	31.71 (1.991)	22.37 (1.211)	-65.51 (1.875)	15.51 (0.98)	11	
S3C	*w;Tub-FRT-STOP-FRT-Gal4,UAS-FLP,UAS-CD8-GFP;H94-Gal4,nSyb-Gal80/UAS-FasII;UAS-GluRIIA*^{RNAi} (muscle 7)	+	0.51 (0.026)	28.02 (1.424)	54.94 (3.767)	-67.79 (2.891)	13.39 (1.554)	10	<0.0001 (****), 0.9982 (ns), <0.0001 (****)
S3D	w^{1118} (muscle 6)	-	0.94 (0.022)	34.98 (1.076)	37.21 (1.981)	-67.09 (2.296)	6.95 (0.316)	9	
S3D	w^{1118} (muscle 6)	+	0.45 (0.019)	31.83 (0.873)	70.33 (4.137)	-63.94 (2.095)	7.21 (0.591)	9	<0.0001 (****), 0.9957 (ns), <0.0001 (****)
S3E	*w;Tub-FRT-STOP-FRT-Gal4,UAS-FLP,UAS-CD8-GFP;H94-Gal4,nSyb-Gal80/UAS-FasII;UAS-GluRIIA*^{RNAi} (muscle 6)	-	0.46 (0.027)	31.94 (1.708)	66.54 (3.732)	-62.87 (1.591)	11.69 (1.065)	11	
S3E	*w;Tub-FRT-STOP-FRT-Gal4,UAS-FLP,UAS-CD8-GFP;H94-Gal4,nSyb-Gal80/UAS-FasII;UAS-GluRIIA*^{RNAi} (muscle 6)	+	0.37 (0.012)	29.38 (0.989)	79.5 (4.575)	-64.48 (1.592)	9.09 (0.855)	10	0.2349 (ns), >0.9999 (ns), 0.3159 (ns)
